# Oil and Gas Wastewater Components Alter Streambed Microbial Community Structure and Function

**DOI:** 10.3389/fmicb.2021.752947

**Published:** 2021-11-29

**Authors:** Denise M. Akob, Adam C. Mumford, Andrea Fraser, Cassandra R. Harris, William H. Orem, Matthew S. Varonka, Isabelle M. Cozzarelli

**Affiliations:** ^1^United States Geological Survey, Geology, Energy & Minerals Science Center, Reston, VA, United States; ^2^United States Geological Survey, Water Mission Area, Reston, VA, United States

**Keywords:** unconventional oil and gas production, class II injection well, wastewaters, microbial activity, microbial communities

## Abstract

The widespread application of directional drilling and hydraulic fracturing technologies expanded oil and gas (OG) development to previously inaccessible resources. A single OG well can generate millions of liters of wastewater, which is a mixture of brine produced from the fractured formations and injected hydraulic fracturing fluids (HFFs). With thousands of wells completed each year, safe management of OG wastewaters has become a major challenge to the industry and regulators. OG wastewaters are commonly disposed of by underground injection, and previous research showed that surface activities at an Underground Injection Control (UIC) facility in West Virginia affected stream biogeochemistry and sediment microbial communities immediately downstream from the facility. Because microbially driven processes can control the fate and transport of organic and inorganic components of OG wastewater, we designed a series of aerobic microcosm experiments to assess the influence of high total dissolved solids (TDS) and two common HFF additives—the biocide 2,2-dibromo-3-nitrilopropionamide (DBNPA) and ethylene glycol (an anti-scaling additive)—on microbial community structure and function. Microcosms were constructed with sediment collected upstream (background) or downstream (impacted) from the UIC facility in West Virginia. Exposure to elevated TDS resulted in a significant decrease in aerobic respiration, and microbial community analysis following incubation indicated that elevated TDS could be linked to the majority of change in community structure. Over the course of the incubation, the sediment layer in the microcosms became anoxic, and addition of DBNPA was observed to inhibit iron reduction. In general, disruptions to microbial community structure and function were more pronounced in upstream and background sediment microcosms than in impacted sediment microcosms. These results suggest that the microbial community in impacted sediments had adapted following exposure to OG wastewater releases from the site. Our findings demonstrate the potential for releases from an OG wastewater disposal facility to alter microbial communities and biogeochemical processes. We anticipate that these studies will aid in the development of useful models for the potential impact of UIC disposal facilities on adjoining surface water and shallow groundwater.

## Introduction

Wastewaters co-produced during shale gas development become of particular concern in the event of an environmental release, potentially affecting surface and shallow subsurface waters and the biological communities that depend on them. Shale gas wastewaters are comprised of a mixture of hydraulic fracturing fluids (HFFs) and water from the fractured formation (Warner et al., 2014; Akob et al., 2015; Rowan et al., 2015). This water is co-produced with natural gas and/or oil and generally contains high total dissolved solids (TDS; up to 350 g/L), along with a variety of chemicals used in the hydraulic fracturing process, additional organic compounds derived from the formation, and naturally occurring radioactive materials (Strong et al., 2013; Engle et al., 2014; Orem et al., 2014; Warner et al., 2014; Akob et al., 2015; Rowan et al., 2015). The magnitude of the challenge of managing this wastewater becomes clear when considering that an average Marcellus Shale gas well produces up to 20 million liters of wastewater over its lifetime; and nearly 10,000 wells are currently active in Pennsylvania, West Virginia, and Ohio (Amico et al., 2011; Ohio Department of Natural Resources [OHDNR], 2017; West Virginia Department of Environmental Protection [WVDEP], 2017). In the Marcellus region, much of this wastewater is disposed of *via* underground injection in United States Environmental Protection Agency [U.S. EPA] (2019) Class II Underground Injection Control (UIC) disposal wells. As of this writing, there are approximately 36,000 Class II UIC wells used for disposal of oil and gas (OG) wastewaters across the United States (United States Environmental Protection Agency [U.S. EPA], 2019); however, only a limited number of studies have investigated the potential impact of underground injection and related activities on surface and near-surface environments (Akob et al., 2016; Kassotis et al., 2016; Orem et al., 2017).

Hydraulic fracturing fluids are composed of a wide range of organic and inorganic components, including gelling agents, scale reducers, biocides, water, and proppants. The fate of these inorganic and organic components in the highly saline matrix of OG wastewaters is not well established, although recent reports indicate that much of the total organic content is biodegradable (Kekacs et al., 2015; McLaughlin et al., 2016; Mouser et al., 2016a; Heyob et al., 2017; Butkovskyi et al., 2018; Hanson et al., 2019; Lozano et al., 2019; Rogers et al., 2019). Ethylene glycol, commonly used in HFF as a scale reducer, is readily biodegraded under aerobic and anaerobic conditions with complete removal observed between 24 h and 28 days (Dwyer and Tiedje, 1983; Kameya et al., 1995; Staples et al., 2001; Mrklas et al., 2004). Under aerobic and anaerobic conditions, ethylene glycol degrades to acetic acid and ethanol, with methane as a final product under anaerobic conditions (Dwyer and Tiedje, 1983; Mrklas et al., 2004). Guar gum is used as a gelling agent in HFF and is reported to degrade under the mixed aerobic/anaerobic conditions present in an activated sludge treatment system (Lester et al., 2014). Campa et al. (2018) saw degradation of the biocide glutaraldehyde, which is often used in HFFs, in stream water-based microcosms. However, studies suggest that the high salinity of shale gas wastewaters can inhibit aerobic biodegradation (Kekacs et al., 2015; McLaughlin et al., 2016; Hanson et al., 2019). While these studies indicate that some organic compounds present in HFF are utilized as carbon sources by indigenous microbes, the potential effect of adding this carbon to the stream environments is less clear. The addition of biodegradable organic carbon to otherwise carbon-limited environments has the potential to drive the reductive dissolution of iron minerals (Love et al., 2014), a process that has been linked to the mobilization of metals, including arsenic (Pedersen et al., 2006; Barringer et al., 2010; Mumford et al., 2012; Cozzarelli et al., 2016). Work by Mumford et al. (2018) observed that addition of biocides decreased iron reduction when added to stream sediment microcosms, although recovery of activity was observed over several weeks of incubation.

The role of shale gas wastewaters on the structure of microbial communities in the shallow subsurface is not well known and is the topic of a considerable amount of current research. Under aerobic conditions, studies found that treatment with synthetic HFF led to an increase in the relative abundance of *Pseudomonas* in soil-groundwater (Kekacs et al., 2015; Mouser et al., 2016b) and river-water (Hanson et al., 2019) microcosms, while anaerobic conditions led to an increase in *Desulfovibrio*. Biodegradation of non-ionic surfactants under anaerobic conditions was associated with an increase in the abundance of *Firmicutes*-affiliated metagenomes (Heyob et al., 2017), while aerobic biodegradation of these compounds was associated with an increase in members of the *Proteobacteria* (Hanson et al., 2019). Lozano et al. (2019) observed shifts in microbial soil communities exposed to different synthetic HFF compositions. Another commonly used biocide in hydraulic fracturing is 2,2-dibromo-3-nitrilopropionamide (DBNPA), which has a short half-life of 4 hours at neutral pH (United States Environmental Protection Agency [U.S. EPA], 1994). DBNPA was observed to affect microbial community structure when added to anaerobic stream-sediment microcosms (Mumford et al., 2018) and aerobic stream-water microcosms (Campa et al., 2019). Campa et al. (2019) also documented the formation of numerous brominated byproducts from DBNPA degradation. Several studies have examined the microbial communities in shale gas wastewaters and OG wastewaters retention ponds (Struchtemeyer et al., 2011; Struchtemeyer and Elshahed, 2012; Murali Mohan et al., 2013a,b; Cluff et al., 2014; Mouser et al., 2016a); however, it remains unknown if these microbes could migrate from surface spills to the shallow subsurface.

Recent work revealed impacts to a stream from activities at a UIC OG wastewater disposal facility in West Virginia, including alterations to stream geochemistry (Akob et al., 2016; Orem et al., 2017) and microbial community dynamics (Akob et al., 2016; Fahrenfeld et al., 2017), and elevated endocrine activity known to disrupt reproduction and/or development in aquatic animals (Kassotis et al., 2016). Alterations in stream chemistry included elevated concentrations of Cl and Sr, indicative of impact from shale gas wastewaters. Analysis of microbial communities from the site showed clear differences in the microbial community structure immediately downstream from the facility compared with upstream. This result suggested that the potential for shale gas wastewaters to influence the microbial communities who are believed to play a crucial role in the fate and transport of both organic and inorganic components of shale gas wastewaters (Akob et al., 2016; Fahrenfeld et al., 2017). Further research using bed sediments from this wastewater facility demonstrated the potential for biocides used in HF fluids to alter anaerobic microbial community structure and inhibit iron reduction (Mumford et al., 2018). While the study described by Mumford et al. (2018) described changes only to the anaerobic microbial community, this study describes the effects in a mixed aerobic/anaerobic system more reflective of streambed conditions. The aim of the microcosm-based experiments was to assess the response of streambed microbial communities to the high TDS brine characteristic of OG wastewaters in combination with a commonly used biocide (DBNPA) and ethylene glycol (a scale reducer). This work provides information on the community-level effects of HFF components in the environment and the potential for microbial activity to be altered in the event of OG wastewater releases into streams.

## Materials and Methods

### Site Description and Sampling

Samples were collected for microcosm studies on June 18, 2014, from an unnamed, first-order stream in the Wolf Creek watershed in West Virginia, United States, that runs through a UIC disposal facility (Supplementary Figure 1). The disposal facility included the disposal well, which injected wastewater to 792.5 m below surface, brine storage tanks, an access road, and (formerly) two small, unlined impoundment ponds as described in Akob et al. (2016). Sediments were collected from two sites along the stream that runs through the disposal facility (Supplementary Figure 1): upstream, background Site 4, and downgradient Site 7 (downstream from former impoundment ponds). Site 4 was not impacted by activities at the site, as indicated by low specific conductance and concentrations of Na, Cl, and other elements (Table 1; Akob et al., 2016; Orem et al., 2017). Site 7 was revealed to be impacted by activities at the site, as seen by elevated specific conductance and increased concentrations of OG wastewater markers relative to the conditions found upstream from the facility (Table 1; Akob et al., 2016; Orem et al., 2017). Although previous studies documented the effects of activities at the UIC disposal facility (Akob et al., 2016; Orem et al., 2017), the actual pathway of contamination was not identified. The authors acknowledged that a number of point sources of contamination could exist, including surface spills or leaking storage ponds or tanks.

**TABLE 1:** Field parameters, non-volatile dissolved organic carbon (NVDOC), and major anion and cation concentrations of Site 4 (background) and Site 7 (impacted) water samples collected in June 2014 in a tributary of Wolf Creek that is adjacent to an oil and gas wastewater disposal facility.

Sample	Type, location	Field parameters			NVDOC (mg/L)	Cl (mg/L)	Na (mg/L)	Ba (mg/L)
		pH	Conductivity (μS/cm)	Fe(II) (mg/L)				
Site 4	Background, upstream from disposal facility	6.47	74.0	0.2	1.13	0.88	6.96	136
Site 7	Downstream from former impoundment ponds	6.36	416	8	2.49	115	63.4	653

*Data from Akob et al. (2016).*

Water for the microcosms was collected at the background Site 4 into sterile polypropylene carboys from the approximate center of the stream using a peristaltic pump (Geopump™ Peristaltic Pump Series II, Geotech Environmental Equipment, Inc., Denver, CO, United States). Water from Site 4 was selected as the medium for the microcosms, as it is located upstream of the disposal facility and flows downstream toward Site 7; therefore, it can be considered representative of the water flowing through the system, but without the effects of the disposal operations. Sediment samples from each site were collected using sterile polypropylene scoops into sterile Whirl-Pak^®^ bags (Nasco, Fort Atkinson, WI, United States) from the upper 5 cm of the streambed. All samples were stored on ice in the field then at 4°C until the start of the experiment. Chemical analyses on water and sediment samples are described in Akob et al. (2016) and Orem et al. (2017), which included analysis of alkalinity, cations, anions, strontium, oxygen and hydrogen isotopes, non-volatile dissolved organic carbon (NVDOC), trace inorganic elements, organic compounds, carbon, nitrogen, and sulfur elemental analysis, Fe speciation, and total inorganic elements. A previous work showed that iron and percent carbon were elevated in Site 7 sediments compared with Site 4, highlighting the differences between the sites due to activities at the disposal facility (Akob et al., 2016).

### Microcosm Design and Construction

Microcosms were constructed on July 7, 2014, to monitor microbial activity and population dynamics in the presence of HFF organic additives in a dilute brine solution composed to simulate a spill of shale gas wastewater. The HFF components chosen were a biocide, DBNPA, and a scale reducing additive, ethylene glycol, as they are commonly used during hydraulic fracturing in the Marcellus Shale region.^1^ Concentrations of DBNPA and ethylene glycol were selected to simulate likely concentrations in wastewater based on a review of commonly used concentrations reported by FracFocus (see text footnote 1). A synthetic wastewater brine was formulated based on the average chemistry of produced waters from the Appalachian Basin as described by Dresel and Rose (2010). The synthetic wastewater brine contained the following per liter: 12 g of CaCl_2_*2H_2_O, 3.1 g of MgCl_2_*6H_2_O, 1.2 g of SrCl_2_*6H_2_O, 22.8 g of NaCl, 0.27 g of KCl, 0.046 g of NaHCO_3_, 0.23 g of NaBr (anhydrous), 0.015 g of MnCl_2_*4H_2_O, and 0.31 g of BaCl_2_*2H_2_O. Five treatment conditions were tested: (1) brine + DBNPA, (2) brine + ethylene glycol, (3) brine, (4) unamended control, and (5) killed control (Table 2). To compare the response of streambed microbial communities that were or were not affected by activities at the UIC facility, sediments from Site 4 (background) and impacted Site 7 were used. All microcosms were incubated under oxic conditions and without shaking to allow for a transition from aerobic to anaerobic conditions within the sediment to mimic the oxic-to-anoxic gradient found in the streambed.

**TABLE 2:** Experimental conditions for the microcosms.

Treatment	Amendment	Sediment source	# replicates	Purpose
Brine + DBNPA	Artificial brine and 8.3 mg/L of DBNPA (biocide)	Background (Site 4)	3	Assess impact of biocide and brine on microbial community structure and metabolism
		Impacted (Site 7)	3	
Brine + ethylene glycol	Artificial brine and 3.7 mg/L of ethylene glycol	Background (Site 4)	3	Assess impact of scale reducer on microbial community structure and metabolism
		Impacted (Site 7)	3	
Brine	Artificial brine	Background (Site 4)	3	Assess impact of brine on microbial community structure and metabolism
		Impacted (Site 7)	3	
Unamended	None (control)	Background (Site 4)	3	Assess impact of culture conditions on microbial community structure and function
		Impacted (Site 7)	3	
Killed control	None (control)	Background (Site 4)	2	Control for abiotic reactions
		Impacted (Site 7)	2	

*Microcosms were constructed using stream sediments collected upstream (Site 4) and downstream (Site 7) of an OG wastewater disposal facility where effects on stream biogeochemistry and microbiology were documented immediately downstream. The composition of artificial brine was designed to mimic the inorganic chemistry of OG wastewaters from the Marcellus region and had a total dissolved solids content of 5,000 mg/L as described in the section “Materials and Methods.”OG, oil and gas; DBNPA, 2,2-dibromo-3-nitrilopropionamide.*

Live microcosms were prepared by adding 200 g of homogenized sediment from either site to an autoclaved 1-L glass bottle (Schott AG, Mainz, Germany), and then 500 ml of 0.2-μm filter-sterilized background site water was added. Water from the background site was used as media to simulate the conditions found at the field site and was filter-sterilized to focus on the response of the sediment-associated microbial communities. Bottles were sealed with GL45 black butyl rubber stopper (Glasgerätebau Ochs, Bovenden, Germany) and GL45 red aperture caps (Schott AG, Mainz, Germany). All live microcosms were prepared in triplicate. Live microcosms were amended with 100 ml of treatment solution. A biocide stock solution was made by dissolving 6.25 g of DBNPA in 250 ml of Milli-Q ultrapure water, which was then filter-sterilized with a 0.2-μm Acrodisc^®^ Supor^®^ membrane syringe filter (Pall Life Sciences, Port Washington, NY, United States). To make the biocide treatment solution, 2 ml of the DBNPA stock solution was added to 1 L of artificial brine. A 2% ethylene glycol stock was made, and then 1 ml of stock was added to 1 L of artificial brine. The initial concentration of DBNPA and ethylene glycol in the microcosms at day 0 was 8.3 and 3.7 mg/L, respectively.

Killed control microcosms were prepared by adding 50 g of homogenized sediment from either site to an autoclaved 250-ml glass bottle (Schott AG, Mainz, Germany), and then 125 ml of filter-sterilized background site water was added prior to sealing as described above. Bottles were autoclaved at 121°C for 20 min to kill sediment microorganisms. Killed control microcosms were prepared in duplicate.

### Microcosm Sampling and Analytical Methods

Over the course of the 94-day incubation, microcosms were sampled for NVDOC, and ethylene glycol, as well as headspace concentrations of methane (CH_4_), carbon dioxide (CO_2_), and oxygen (O_2_). Samples of headspace gas were taken and analyzed for oxygen, carbon dioxide, and methane every 1–7 days, and exact sampling time points are provided in Akob et al. (2021). Liquid samples for analysis of NVDOC were collected on days 0, 14, 28, and 85. Sulfate and nitrate were below reported limits of 0.05 and 0.25 mM, respectively, at day 0 and not measured at later time points. Wet chemical extractions were used to simultaneously determine aqueous Fe(II) and HCl-extractable Fe(II) in sediment. Microcosm slurry samples were collected at days 1, 14, 22, and 55 and then extracted in 0.5 M HCl for 1 h; then extracts were quantified colorimetrically by analysis in ferrozine buffer (50 mM HEPES, 0.1% ferrozine, pH 7) (Kostka and Luther, 1994). Absorbance was measured at 562 nm, and iron concentrations were calculated based on a standard curve of known Fe(II) concentrations.

Methane, CO_2_, and O_2_ were measured using gas chromatography (GC) according to methods described in Cozzarelli et al. (2017) and Shelton et al. (2020). Headspace samples were collected from microcosms using a sampling valve and pressure lock, and gas tight syringes (Valco Instruments Co. Inc., Houston, TX, United States) with a non-coring needle. The sampling valve was composed of a Hamilton HV Plug Valve (Hamilton Company, Reno, NV, United States), sealed with Thermogreen™ LB-2 5-mm septa (Supelco, Bellefonte, PA, United States), and Kel-F^®^ female and male luer fittings (Hamilton No. 35031 and No. 35030, Hamilton Company, Reno, NV, United States). A sterile syringe needle was attached to the male end and then inserted into a flamed stopper of a microcosm bottle. With the use of a pressure lock syringe, a volume of gas was removed and then injected into a HP6890 GC (Hewlett Packard HP 6890 Series GC—GMI Inc.). Gasses were separated on a Haysep N 80–100 mesh column with a 3-m-long, 1/8-inch internal diameter Nafion Dryer and analyzed with a thermal conductivity detector (TCD). The GC was operated with nitrogen as the carrier gas (20 ml min^–1^ total flow) at temperatures of 40, 155, and 180°C for the oven, inlet, and detector, respectively, and an injector (constant makeup) flow rate of 20 ml min^–1^ total flow. GC signals were analyzed using Class VP 7.3 software (Shimadzu, Columbia, MD, United States). Instrument responses were standardized using mixed CO_2_/O_2_ standards (Cal Gas Direct Inc., Huntington Beach, CA, United States) and CH_4_ standards (SCOTT™ Specialty Gases, Plumsteadville, PA, United States). Gas pressure was measured in tubes using a GMH 3111 digital pressure meter with a GMSD needle pressure transducer (Greisinger Electronic, Regenstauf, Germany). Concentrations in parts per million (ppm) from the GC were converted to μmol of gas in headspace using Eq. (1):


(1) $$\frac{\left(P_{atm}+P_{HS}\right)\times V_{HS}}{R\left(T+273.15\right)}\text{×}\frac{X_{CH4}}{10^{6}}=n_{CH4}$$

where *X*_*CH*4_ is the concentration of methane in headspace in ppm, *V*_*HS*_ is the headspace volume (ml), *P*_*HS*_ is the headspace overpressure in millibar, *P*_*atm*_ is the room pressure in mbar, *T* is the lab temperature in °C, and *R* is the ideal gas constant 8.314 × 10^4^ (ml mbar mole^–1^ K^–1^).

Samples collected for NVDOC were filtered through 0.20-μm Supor^®^ filters (Pall, Port Washington, NY, United States) into baked amber-glass VOA vials with Teflon^®^ -faced septa and then preserved with hydrochloric acid (HCl). NVDOC concentrations were analyzed by high-temperature combustion using a TOC-Vcsn Total Organic Carbon Analyzer (Shimadzu Corporation, Kyoto, Japan).

Microcosm samples (unfiltered) for ethylene glycol determinations were stored frozen at −20°C in 50-ml Falcon^®^ tubes (Corning Inc., Corning, NY, United States) until analysis. DBNPA concentrations could not be measured. Concentrations of ethylene glycol were determined after conversion to benzoyl esters by high-performance liquid chromatography (HPLC) analysis, with UV and electrospray ionization mass spectrometric detection after Holčapek et al. (1999). In this study, 1 ml of solution from the microcosms was added to a clean (baked at 450°C) glass test tube with Teflon^®^-lined cap; and internal standards were added to the sample in the test tube: 30 μl of benzyl alcohol (from 200 mg/L of stock solution) and 100 μl of phenol (from 500 mg/L of stock solution). Then, 700 μl of 30% NaOH solution and 20 μl of benzoyl chloride (liquid, ACS reagent grade 99%) were added to the test tube, and the solution was shaken in the capped test tube for 10 min, producing benzoyl esters of the ethylene glycol under the alkaline conditions. After shaking, 1 ml of pentane was added, and the test tube was again capped and shaken for 10 min. The pentane extract layer (0.7 ml) containing the benzoyl esters of ethylene glycol was then removed using an auto-pipette and placed in a clean glass test tube. An additional 1 ml of pentane was added to the sample mixture and shaken for 10 min; 1 ml of the pentane layer was then removed by auto-pipette and combined with the first extract. The combined pentane extract was dried using a NitroVap (Parker Hannifin, Hemel Hempstead, United Kingdom), and the residue was dissolved in 100 μl of 55% acetonitrile in Milli-Q water. A working standard solution was prepared by diluting a stock standard (500 mg/L of ethylene glycol) 10×. Analytical standards (1, 2.5, 5, 10, and 20 mg/L) were prepared from the working standard and analyzed like the samples to produce a standard curve. Blanks that consisted of Milli-Q water were run in the same manner as the samples and standards. HPLC was used to determine the presence and concentrations of the glycol benzoyl ester derivative and an internal standard. An aliquot (25 μl) of extract was injected into a stream of 55% (v/v) acetonitrile/Milli-Q water at a flow rate of 1.0 ml/min. The injected extract was pumped through a reversed-phase column (Waters NovaPak C-18, 150 mm × 3.9 mm; Waters Corporation, Milford, MA, United States) and passed through a diode array spectrophotometric detector (Waters 996 Photodiode Array Detector) where the light absorbances of the individual bands were determined at the time they passed through the detector. Absorbances of 190–300 nm were recorded at the retention times of the derivative and the internal standards. However, only the absorbance value at 237 nm was used for quantification. The peak area ratios of the sample benzoyl ester/internal standards were calculated and reported. Peaks were also followed by Micromass ZQ 2,000 mass spectrometer, but the mass spectrometer peaks were used for compound confirmation, not for quantification. The method had a detection limit of 1 mg/L of ethylene glycol, and relative SD ranged from 2 to 4% (Holčapek et al., 1999).

Statistical analyses of geochemical data were performed using Prism version 8 and version 9 (GraphPad Software, San Diego, CA, United States). Graphs were generated using Prism version 8. Significant differences in concentrations of analytes in microcosms were tested using unpaired *t*-tests with Welch’s correction. *P* values of <0.05 were considered significant.

### Microbial Community Analysis

Subsamples of sediment used to construct the microcosms were frozen to provide information on the starting microbial community (day 0). At 14 days of incubation, microcosms (total of 24 bottles) were shaken, and then 20 ml of sediment slurry samples was collected using sterile syringes for microbial community analysis. Slurries were centrifuged at 5,000 × *g*, then the supernatant was removed, and the pellet was stored at −80°C prior to DNA extraction. DNA was extracted from triplicate day 0 samples and day 14 microcosm samples using the Mo Bio PowerMax^®^ DNA Isolation Kit (MO-BIO Laboratories, Carlsbad, CA, United States) according to the manufacturer’s protocol. DNA extracts were sent to Michigan State University’s Research Technology Support Facility (RTSF, East Lansing, MI, United States) for Illumina 16S iTag sequencing (Illumina, Inc., San Diego, CA, United States). Amplicon libraries of the V4 hypervariable region of the 16S rRNA gene were prepared using dual indexed, Illumina compatible primers 515f and 806r (Apprill et al., 2015; Parada et al., 2016; Walters et al., 2016) following the protocol developed by the Patrick Schloss lab (Kozich et al., 2013). Following PCR, all products were batch normalized using Invitrogen SequalPrep DNA Normalization (Invitrogen, Carlsbad, CA, United States) plates, and products were recovered from the plate pooled. Pooled amplicons were cleaned up using AmpureXP magnetic beads 0.8× (vol/vol) beads/pool ratio (Beckman Coulter, Indianapolis, IN, United States). This pool was loaded onto an Illumina MiSeq Standard v2 flow cell, and sequencing was performed in a 2 × 250 bp paired-end format using a 500-cycle v2 reagent cartridge. Custom sequencing primers were added to appropriate wells of the reagent cartridge as described in Kozich et al. (2013). Base calling was done by Illumina Real-Time Analysis (RTA) v1.18.54, and output of RTA was demultiplexed and converted to FastQ format with Illumina Bcl2fastq v2.19.1.

Microbial sequence data were processed for quality control and alignment, and taxonomic assignment using MOTHUR v1.39.5 (Schloss et al., 2009; Kozich et al., 2013; Schloss, 2015) using the United States Geological Survey (USGS) Advanced Research Computing (ARC) Yeti high-performance computing facility. Operational taxonomic units (OTUs) were assigned based on a 97% similarity cutoff, with taxonomy assigned based on similarity the Silva nr99 v128 database (Pruesse et al., 2007; Quast et al., 2012). Taxonomic assignment for all OTUs is available as a BIOM file in Akob et al. (2021). The mothur batch script used for sequence processing is presented in the Supplementary Material. The phylogenetic affiliation of OTUs were converted to percent (%) relative abundance and figures presented the results as averages for triplicate samples. Percent (%) relative abundance are presented in Supplementary Table 3 for each sample and as average ± standard deviation for each treatment. Taxa relative abundance data were plotted using Prism version 9 (GraphPad Software, San Diego, CA). Maximum likelihood trees for weighted UniFrac (Lozupone et al., 2011) analysis were constructed using RAxML (Stamatakis, 2014) and ExaML (Kozlov et al., 2015).

Data analysis was performed in R, using core components and the vegan, phyloseq, and dplyr packages (McMurdie and Holmes, 2013; Okansen et al., 2014; R Core Team, 2015; Oksanen et al., 2018; Wickham et al., 2018); the code used for processing is presented in the Supplementary Material. For multivariate analysis of microbial community structure, the sequence data were first randomly subsampled to even depth, with the number of subsamples set to equal the smallest number of sequences within a sample set, using the ‘rarefy_even_depth’ function from the phyloseq R package (McMurdie and Holmes, 2013). A weighted UniFrac (Lozupone et al., 2011) distance matrix was constructed from this resampled data set using the ‘phyloseq:distance’ function in phyloseq. Non-metric multidimensional scaling (NMDS) constrained to 2 axes was performed on this distance matrix, with a minimum of 1000 replicates performed using the ‘metaMDS’ function in the vegan R package (Okansen et al., 2014). Geochemical parameters were fit to the NMDS plots as vectors using the ‘envfit’ function in vegan; only vectors with *p*-values <0.05 are shown. 95% confidence intervals on NMDS plots were generated using the ‘ordiellipse’ function in vegan. Differential abundance analysis was performed using DESeq2 (Love et al., 2014) as described in the Supplementary Material “R Analysis Code”. Results are presented as log_2_fold changes and are significant at a *p*-value < 0.05. Bar graphs of the log_2_fold change were generated using Prism version 9 (GraphPad Software, San Diego, CA).

## Results and Discussion

### Changes in Metabolic Processes in Sediment Microcosms

#### Background Site Microcosms

**FIGURE 1:**
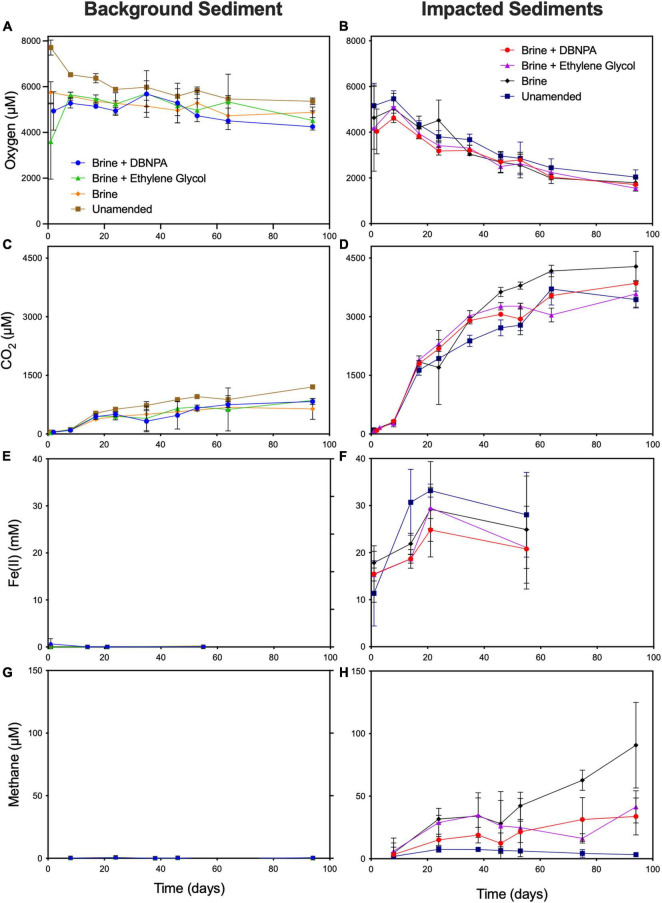
Concentrations of oxygen **(A,B)**, CO_2_ **(C,D)**, Fe(II) **(E,F)**, and methane **(G,H)** in microcosms constructed with sediments from background and impacted sites. Microcosms were amended with a high TDS brine with or without DBNPA (biocide) and ethylene glycol or left unamended as controls. Results are averages ± SDs for triplicate microcosms. Intermediate data points were omitted for clarity but are available in Akob et al. (2021). TDS, total dissolved solids; DBNPA, 2,2-dibromo-3-nitrilopropionamide.

In the unamended background microcosms, the headspace oxygen concentration dropped significantly (7,540 ± 270 μM on day 1 to 6,040 ± 33.2 μM on day 15, *p* < 0.05) over the first 15 days of incubation as well as between the 15th and 24th days of incubation (6,040 ± 33 μM on day 15 to 5,850 ± 52.8 μM on day 24, *p* < 0.05) (Figure 1A). Oxygen consumption from the 24th to 53rd day of incubation was minimal (*p* < 0.05), suggesting a shift to anaerobic metabolism despite oxygen remaining in the headspace. The headspace CO_2_ concentration increased significantly between the first and 15th days of incubation (48.7 ± 0.2 μM on day 1 to 552 ± 21.2 μM on day 15, *p* < 0.05), did not change significantly between the 15th and 24th days of incubation (552 ± 21.2 μM to 648 ± 18.5 μM *p* < 0.05), and then increased significantly between the 24th and 53rd days of incubation (648 ± 18.5 μM to 968 ± 27.3 μM *p* < 0.05) (Figure 1C). These changes in CO_2_ concentration without continued oxygen consumption provide further indication of a shift from aerobic to anaerobic respiration or a shift in community structure to utilize different carbon sources. As shown in Figure 1C, the unamended microcosms produced more CO_2_ than any of the amended microcosms with an average headspace concentration of 956 μM of CO_2_ after 53 days of incubation. This result was significantly (*p* < 0.05) greater than that in the brine- (612 μM of CO_2_), brine + DBNPA- (666 μM of CO_2_), or brine + ethylene glycol-amended (694 μM of CO_2_) microcosms.

**FIGURE 2:**
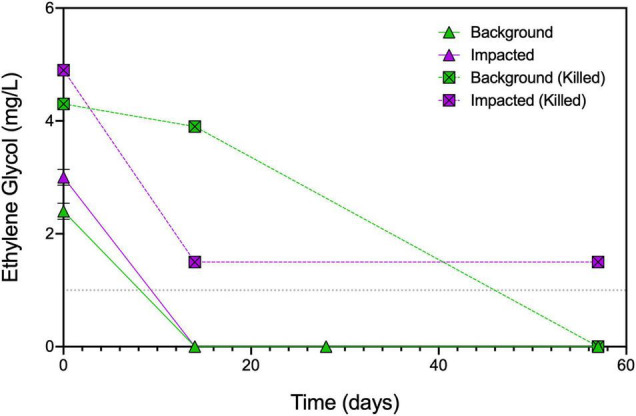
Concentrations of ethylene glycol over time in select microcosms. Duplicate samples were measured for live impacted and background sediment microcosms at days 0, 14, 28, and 57 ± SDs. Single replicate samples for killed impacted or background sediment microcosms were measured at days 0, 14, and 57. Dashed line indicates the method detection limit of 1 mg/L of ethylene glycol; all data are presented in Supplementary Table 1.

Brine + ethylene glycol amendment led to the production of slightly more CO_2_ than brine alone (*p* = 0.02), and ethylene glycol concentrations decreased from 2.4 ± 0.14 mg/L to below the detection limit within 14 days of incubation (Figure 2). While the degradation of ethylene glycol alone is insufficient to explain the increase in CO_2_, it has been shown to increase the bioavailability of iron as a terminal electron acceptor (Mumford et al., 2018). The concentration of ethylene glycol in killed control microcosms changed minimally over the first 14 days of the experiment (day 0 = 4.3 mg/L and day 14 = 3.9 mg/L; Supplementary Table 1), suggesting that the disappearance of ethylene glycol in the live microcosms was due to microbial degradation. Taken together, these results indicate that the introduction of brine has the greatest effect on respiration in sediments not known to have been previously exposed to elevated TDS. This observation is in line with previous studies that observed inhibition of aerobic biodegradation due to the high salinity of shale gas wastewaters (Kekacs et al., 2015; McLaughlin et al., 2016; Hanson et al., 2019).

Amendment with brine, brine + DBNPA, or brine + ethylene glycol appeared to limit oxygen consumption; there was no significant change in headspace oxygen concentration over the entire course of the incubations (*p* > 0.05, Figure 1A). Although there was no significant loss of oxygen in the brine + DBNPA-amended microcosms, we did see a slight decrease in oxygen when comparing between the 15th and 24th days and the 24th and 53rd days of incubation, suggesting that the biocide may have given a competitive advantage to aerobic organisms. The NVDOC data appear to be consistent with the selection for aerobic respiration in the brine + DBNPA treatment, as concentrations of NVDOC remained high at day 28, while in the other microcosms, NVDOC had measurably decreased (Supplementary Figure 2A). No detectable Fe(II) or methane was produced in any of the microcosms prepared using sediment from the background site (Figures 1E,G). This result was not surprising given that in previous studies, *in situ* background sediment microbial communities from this site were observed to be dominated by aerobic taxa (Akob et al., 2016; Fahrenfeld et al., 2017).

In the killed microcosms, no oxygen consumption was observed in any of the killed control treatment bottles over the first 40 days of incubation (Supplementary Figure 3A). Between 38 and 43 days of oxygen decreased in the brine-amended microcosms, which was also associated with a decrease in headspace carbon dioxide (Akob et al., 2021), which may be due to abiotic factors such as iron mineral oxidation.

#### Impacted Site Microcosms

In the microcosms prepared with sediment from the impacted site, headspace oxygen concentrations did not decrease significantly in the unamended, brine, brine + DBNPA, or brine + ethylene glycol treatments over the first 15 days of incubation (Figure 1B). Between the 15th and 24th days of incubation, significant decreases in headspace oxygen concentration were observed in the microcosms treated with brine + DBNPA (*p* < 0.05) and brine + ethylene glycol (*p* < 0.05), suggesting that the microbial community adapted to utilize the oxic headspace. Between days 24 and 53 of incubation, significant losses of headspace oxygen were observed in the brine (*p* = 0.03) and brine + ethylene glycol (*p* < 0.05) microcosms. Significant CO_2_ production was observed between all of these time points (*p* < 0.05), indicating an active aerobic community (Figure 1D).

Ethylene glycol concentrations also decreased between days 0 and 14 in the impacted microcosms going from 3 ± 0.14 mg/L to below detection (Figure 2). Surprisingly, ethylene glycol decreased from 4.9 to 1.5 mg/L in the killed controls between days 0 and 14, potentially due to abiotic interactions such as sorption to the sediment or residual microbial activity remaining after autoclaving. The differences in the killed controls between the background and impacted sediments are unsurprising given the higher sediment percent carbon (% C) in the impacted sediment (3.2% C in impacted sediment vs. 1.4% C in background sediment) (Akob et al., 2016). In the killed microcosms, oxygen consumption was not observed (Supplementary Figure 3B), but headspace carbon dioxide increased over the first 14 days (Akob et al., 2021), which suggests the potential for residual microbial activity despite autoclaving the microcosms three times.

As shown in Figure 1F, iron reduction was highly variable within each set of triplicate impacted sediment microcosms, as indicated by the large error bars. Following 21 days of incubation, the average Fe(II) concentration of the unamended microcosms increased by approximately 21 μM. In contrast, the Fe(II) concentration increased by 11 μM in the brine-amended microcosm and by 14 μM in the ethylene glycol + brine-amended treatment. Fe(II) in the brine + DBNPA treatment increased by 9 μM, significantly less than was observed in the unamended control (*p* < 0.05). This may indicate a decrease in iron reduction in the presence of DBNPA, which is in line with the lag in the onset of Fe(II) reduction related to DBNPA reported by Mumford et al. (2018) and suggests that this biocide may inhibit iron-reducing organisms. No significant differences were observed when comparing between the three amended microcosm regimes, suggesting that the brine amendment may have the strongest effect on depressing iron reduction.

All amended impacted-sediment microcosms (addition of brine with or without shale gas production additives) led to an increase in CH_4_ production when compared with the unamended controls, indicating that methanogens were favored under elevated TDS conditions (Figure 1H). An increase of 6 μM in headspace CH_4_ was observed within the first 8 to 24 days, which remained steady for the remainder of the experiment. After 53 days of incubation, the microcosms amended with brine generated 42 μM of CH_4_, significantly more than was generated in the unamended microcosms (*p* < 0.05, methane was not detected in the unamended microcosms) and the brine + DBNPA microcosms (*p* < 0.05, 24.5 μM). The effect of amendment with brine + ethylene glycol on methanogenesis is less clear, as one of the three bottles in this treatment had a headspace concentration of 51.68 μM of CH_4_, while the other two bottles had headspace concentrations of 12.94 and 10.05 μM. NVDOC concentrations were markedly higher in the impacted sediment microcosms than in the background sediment microcosms and increased over the first 2 weeks of incubation from ∼10 to ∼20 mg/L of C (Supplementary Figure 2). For the remainder of the incubation time, NVDOC concentrations did not change.

The increase in methanogenesis in the brine-amended microcosms can be attributed at least in part to a significant (*p* < 0.05) 2.6-fold increase (compared with unamended microcosms) in Methanosarcinaceae, a methanogenic family with halophilic members (Oren, 2014). Treatment with brine + DBNPA did not result in a significant change in these organisms in comparison with brine alone, although it did result in a significant (*p* < 0.05) 2.03-fold increase in Methanosaetacae (Supplementary Table 2). Both Methanosarcinaceae and Methanosaetacae are members of the Methanomicrobia, which have been identified in Marcellus flowback impoundments (Murali Mohan et al., 2013b). These findings provide further evidence to support the importance of these organisms to carbon cycling in environments impacted by hydraulic fracturing wastes. Amendment with brine + ethylene glycol did not result in significant changes in the abundances of any known methanogens in comparison with brine alone.

### Changes in Microbial Community Structure

#### Microbial Community Alterations in the Background Microcosms

**FIGURE 3:**
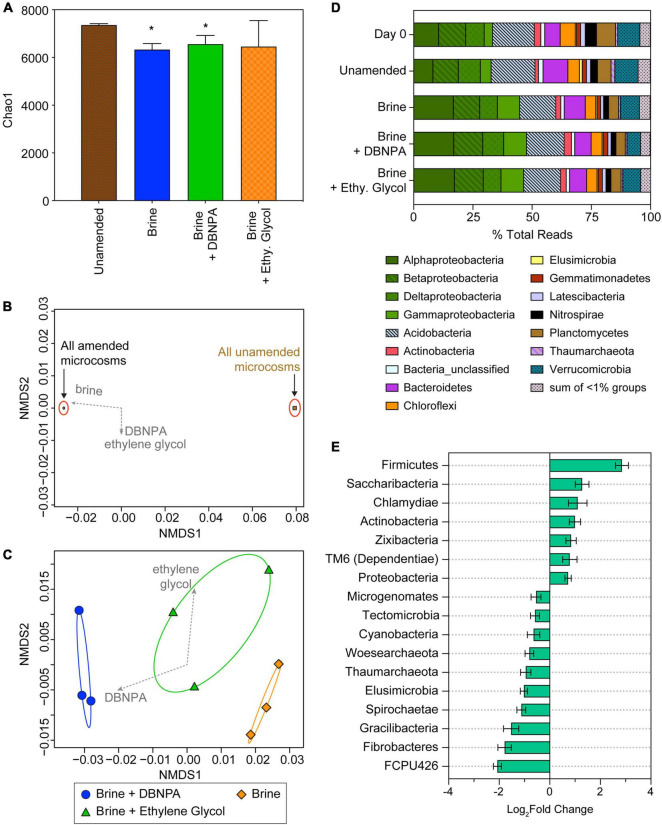
Microbial community composition in background microcosms at day 14 and day 0 sediments. Day 0 sediment was used to construct the microcosms and reflects the starting composition of the microbial community. Microcosms were either amended with brine, brine + DBNPA (biocide), or brine + ethylene glycol (friction reducer; “Ethy. Glycol”) or left unamended as a control. **(A)** Plot of Chao1 diversity indices based on rarefied data (values are averages ± SDs for triplicate microcosms). Samples significantly different from the unamended microcosms are marked with asterisk (*). **(B,C)** NMDS plot based on a weighted UniFrac distance matrix for all background microcosms at day 14 showing divergence between microbial communities in the different treatments. **(B)** The significant divergence of microbial communities from unamended compared with the treatment microcosms, suggesting that the addition of brine is predominantly responsible for much of the shift in community structure. **(C)** The results of an NMDS analysis of amended microcosms, showing the additive effect of DBNPA to brine in shifting the microbial community composition. Envfit vectors (gray) show the contributions of the treatments to shifts in community structure, and ellipses represent 95% CIs. Each point represents a single replicate microcosm for each treatment. **(D)** Relative abundance of taxa at the phyla level except for the Proteobacteria, which are plotted at the class level. Taxa at <1% relative abundance in all samples are listed in Supplementary Table 3A. Data presented are the average relative abundance of triplicate samples; values for each replicate are presented in Supplementary Table 3B. **(E)** Plot of phyla differing significantly (*p* < 0.05) between unamended control and treatment microcosms based on DESeq2 analysis. All treatments were combined and compared with the unamended controls. Bars to the right indicate an increase in abundance in the treatments, while bars to the left indicate a decrease compared with the unamended microcosms. Error bars represent the standard error. NMDS, non-metric multidimensional scaling; DBNPA, 2,2-dibromo-3-nitrilopropionamide.

Microbial community dynamics in the microcosms was investigated at day 14 of incubation, as this was in the time frame of significant changes in geochemistry and metabolic byproducts such as Fe(II) and CO_2_. Microbial diversity, based on the Chao1 diversity metric, decreased between the three treatments (brine, brine + DBNPA, and brine + ethylene glycol) and the unamended background microcosms (Figure 3A). Amendment with brine and brine + DBNPA significantly reduced the diversity (*p* < 0.05). Amendment with brine + ethylene glycol also led to a decrease in Chao1 diversity, although this reduction was not significant (*p* = 0.22) due to the variability in Chao1 scores between the triplicate bottles. When comparing between the treatments, no significant differences in diversity were observed. As all three treatments had brine present, this result suggests an important role for salinity in loss of microbial diversity. Previous studies also saw a decrease in diversity when DBNPA alone was added to sediments (Mumford et al., 2018) and stream water (Campa et al., 2019) collected from streams was not affected by OG-related operations. Our results are consistent with the findings of Mumford et al. (2018) and Campa et al. (2019) which saw that the OG wastewater components of brine and DBNPA can have similar effects on microbial diversity either alone or when added together.

Analysis of the community structure using non-metric multidimensional scaling (NMDS) analysis of a weighted UniFrac distance matrix showed that microbial communities in the amended microcosms significantly differed from those in the unamended controls (Figure 3B). This difference between the communities is seen on Axis 1 with the unamended microcosms clustering together at approximately 0.08 and all of the amended conditions clustering together at approximately −0.02. The amendments were fit to the NMDS scores using the “envfit” function in R, which showed that the only significant variable was amendment with brine (*r*^2^ = 1, *p* = 0.04; Figure 3B). This finding supports the hypothesis that brine is the most significant driver of microbial community structure in this system. Among the brine-amended microcosms, addition of DBNPA had a significant relationship to changes in community structure (*r*^2^ = 0.81, *p* < 0.05), while addition of ethylene glycol did not (*r*^2^ = 0.43, *p* = 0.19) (Figure 3C).

Background sediment (day 0) and microcosm (day 14) microbial communities were dominated by members of the Proteobacteria (Alpha-, Beta-, Delta-, and Gammaproteobacteria classes), Acidobacteria, Bacteroidetes, and Verrucomicrobia (Figure 3D). The day 0 sample is of the sediment used to construct the microcosms and is representative of the starting community composition. Day 0 sediment and unamended microcosm communities following 14 days of incubation did not significantly differ in their taxonomic composition (*p* = 0.67). This result suggests that the incubation conditions alone had a minimal effect on community composition and that the communities in the experiments are representative of what could occur *in situ*. Acidobacteria were the most abundant organisms in the day 0 and unamended microcosm samples representing 18.1 ± 0.6% of total reads (Supplementary Table 3). The next most abundant taxa were the Betaproteobacteria (11.1± 0.5% of total reads), Verrucomicrobia (9.61 ± 0.4% of total reads), and Alphaproteobacteria (9.43± 1.7% of total reads). Deltaproteobacteria and Bacteroidetes represented 8.57 ± 1.1% and 8.51± 2.8% of total reads, respectively.

The community composition among the three amended microcosms (brine, brine + DBNPA, and brine + ethylene glycol) were not significantly different from one another (*p* = 0.93), further indicating that addition of brine was a major driver of microbial community structure. In the amended microcosms, the most abundant taxa were the Alphaproteobacteria at 17.1 ± 0.2% of total reads, followed by Acidobacteria with 15.6 ± 0.3% of total reads, and the Betaproteobacteria at an abundance of 11.8 ± 0.6% of total reads (Supplementary Table 3). Members of the Gammaproteobacteria represented 9.56 ± 0.1% of total reads in all of the amended microcosms. The Deltaproteobacteria, Bacteroidetes, and Verrucomicrobia were observed at similar relative abundance in the amended microcosms (7.93 ± 0.8%, 7.67 ± 1.1%, and 7.14 ± 1.2% of total reads, respectively).

As amendment with brine was associated with the most changes in community composition, we conducted further analysis of the community structure with DESeq2 (Love et al., 2014) to compare the unamended microcosm communities with those in the amended microcosms. The microbial community data for the amended microcosms (brine, brine + DBNPA, and brine + ethylene glycol) were combined into a single parameter, as described in the Supplementary Material, for comparison with the unamended microcosm communities. A total of seven phyla increased in differential abundance, while 10 phyla decreased when comparing the unamended with amended microcosms (*p* < 0.05; Figure 3E). Members of the Firmicutes had the highest increase in differential abundance of all the taxa in the background-amended microcosms (Figure 3E). Within the Firmicutes, three families within the order Clostridiales had significant log_2_fold increases (*p* < 0.05), while only a single family in that order had a log_2_fold decrease (Supplementary Table 2). Interestingly, an increase in the relative abundance of the Alpha- and Gammaproteobacteria was observed (Figure 3D) and likely contributed to log_2_fold increase in the Proteobacteria seen *via* analysis with DESeq2 (Figure 3E). Within the Alphaproteobacteria, 27 families had significant changes (*p* < 0.05) in their differential abundance, including 11 families within the order Rhizobiales that had a log_2_fold increase (Supplementary Table 2). Within the Gammaproteobacteria, 10 families had significant changes (*p* < 0.05), but only four increased. The Betaproteobacteria had a total of seven families that had significant changes (*p* < 0.05), including four families with a log_2_fold increase related to Burkholderiales. Actinobacteria also had an increase in differential abundance in the background-amended microcosms with 15 families significantly increasing (*p* < 0.05) and one family significantly decreasing in abundance (Supplementary Table 2). One Actinobacteria family that significantly increased in differential abundance was the Micrococcaceae, a predominantly aerobic family (Dastager et al., 2014) that was also seen to increase in abundance in DBNPA-amended stream microcosms (Campa et al., 2019). While the percent relative abundance of the members of the Deltaproteobacteria did not vary across all amended microcosms (Figure 3D), we observed a decrease in 13 families all belonging to the order Myxococcales (Supplementary Table 2). Bacteroidetes and Verrucomicrobia both had a decrease in percent of total reads from day 0 and unamended to the three treatments. Within Bacteroidetes, 10 families significantly (*p* < 0.05) decreased, including five belonging to Sphingobacteriales, while Verrucomicrobia had four families with a significant log_2_fold decrease (Supplementary Table 2). The majority of taxa that decreased in differential abundance (Figure 3E) in all of the amended microcosms were members of candidate phyla (FCPU426, Elusimicrobia, Gracilibacteria, Microgenomates, and Woesarchaeota), which may indicate that these taxa are sensitive to the amendments used in this study (e.g., brine, DBNPA, and ethylene glycol). However, three candidate phyla (Zixibacteria, Saccharibacteria, and Dependentiae) increased in differential abundance.

#### Microbial Community Alterations in the Impacted Microcosms

**FIGURE 4:**
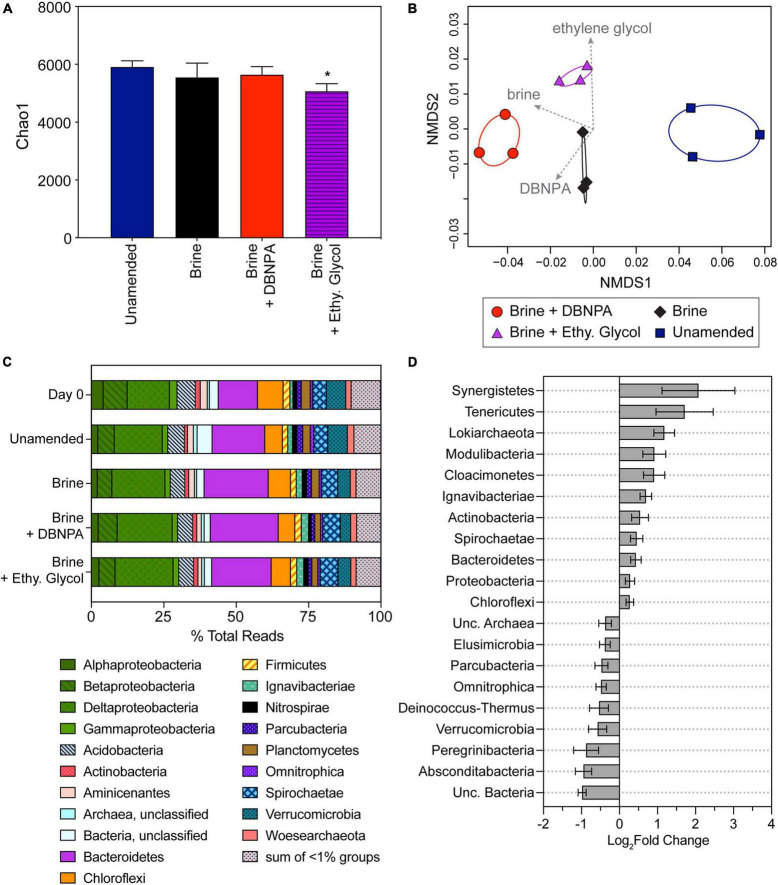
Microbial community composition in impacted microcosms at day 14 and day 0 sediments. Day 0 sediment was used to construct the microcosms and reflects the starting composition of the microbial community. Microcosms were either amended with brine, brine + DBNPA (biocide), or brine + ethylene glycol (friction reducer; “Ethy. Glycol”) or left unamended as a control. **(A)** Plot of Chao1 diversity indices based on rarefied data (values are averages ± SDs for triplicate microcosms). Samples significantly different from the unamended microcosms are marked with asterisk (*). **(B)** NMDS plot based on a weighted UniFrac distance matrix for all impacted microcosms at day 14 showing divergence between microbial communities in the different treatments. Envfit vectors (gray) show the contributions of the treatments (brine, brine + DBNPA, and brine + ethylene glycol) to shifts in community structure, and ellipses represent 95% CIs. Each point represents a single replicate microcosm for each treatment. **(C)** Relative abundance of taxa at the phyla level except for the Proteobacteria, which are plotted at the class level. Taxa at <1% relative abundance in all samples are listed in Supplementary Table 3A. Data presented are the average relative abundance of triplicate samples; values for each replicate are presented in Supplementary Table 3B. **(D)** Plot of phyla differing significantly (*p* < 0.05) between unamended control and treatment microcosms based on DESeq2 analysis. All treatments were combined and compared with the unamended controls. Bars to the right indicate an increase in abundance in the treatments, while bars to the left indicate a decrease compared with the unamended microcosms. Error bars represent the standard error. NMDS, non-metric multidimensional scaling.

A significant decrease in microbial diversity was only seen for the impacted microcosms amended with brine + ethylene glycol when compared with the impacted unamended microcosms (Figure 4A). This was in contrast to the background microcosms, where significant changes in diversity were seen in all three of the amended treatments in comparison with the background unamended microcosm. No significant differences in diversity were observed among the brine- and brine + DBNPA-amended microcosms. This observation could be due to a greater impact of high salts on the community compared with addition of DBNPA or that concentrations of DBNPA were too low to cause an effect. DBNPA is documented to have a short half-life (United States Environmental Protection Agency [U.S. EPA], 1994) and its degradation can be influenced by the presence of organics in the sediment (Blanchard et al., 1987). NMDS analysis of the community structure showed that the amendments altered the community structure (Figure 4B). A significant relationship (*p* = 0.01) was observed between amendment with brine compared with the unamended controls. In addition, limited associations were also seen with the addition of brine + DBNPA (*r*^2^ = 0.5, *p* = 0.06) and brine + ethylene glycol (*r*^2^ = 0.66, *p* = 0.01) compared with the brine-only amendment. The shift in response to brine is noteworthy in that these shifts occurred despite the sediment having been previously exposed to elevated TDS due to inputs of OG wastewater (see Akob et al., 2016; Mumford et al., 2018).

Impacted sediment (day 0) and microcosm (day 14) microbial communities were dominated by members of the Proteobacteria (Alpha-, Beta-, Delta-, and Gammaproteobacteria classes), Actinobacteria, Bacteroidetes, Firmicutes, Planctomycetes, Spirochaetae, and Verrucomicrobia (Figure 4C). Similar to the background samples, impacted day 0 sediment and unamended microcosm community composition did not significantly differ (*p* = 0.75). Bacteroidetes and Deltaproteobacteria were the most abundant organisms in the day 0 and unamended microcosm samples representing 15.8 ± 3.3% and 15.6 ± 1.3% of total reads, respectively (Supplementary Table 3). The next most abundant taxa were Chloroflexi (7.50 ± 2.0% of total reads). Betaproteobacteria, Acidobacteria, and Verrucomicrobia were observed at a similar relative abundance in the day 0 and unamended microcosms (6.98 ± 1.8%, 6.04 ± 0.4%, and 6.71 ± 0.02% of total reads, respectively).

The composition of the microbial communities in the three amended microcosms with impacted sediment was not significantly different from one another (*p* = 0.53). Members of the Bacteroidetes were the most abundant, representing 22.1 ± 1.5% of total reads. The Deltaproteobacteria were the next most abundant taxa in the amended microcosms (19.1± 0.9% of total reads). The Deltaproteobacteria contain many known anaerobic taxa as well as taxa known to reduce iron; and their increased abundance is likely linked to the observed shifts from aerobic to anaerobic metabolic processes. Betaproteobacteria, Acidobacteria, Chloroflexi, and Spirochaetae were observed at similar relative abundances among the amended microcosms (5.72 ± 0.8%, 5.09 ± 0.1%, 6.70 ± 1.0%, and 5.93 ± 0.3% of total reads).

A total of 11 phyla increased in differential abundance, while nine phyla decreased when comparing the unamended with the three amended microcosms using DESeq2 (*p* < 0.05; Figure 4D). Bacteroidetes increased in relative abundance from day 0 and unamended microcosms to the three amended microcosms, contributing to their significant increase in differential abundance (*p* < 0.05). There were 20 Bacteroidetes families with a significant log_2_fold change, including five Sphingobacteriales families and four Bacteroidales families that had a log_2_fold increase (Supplementary Table 2). The Deltaproteobacteria followed a similar pattern to the Bacteroidetes, contributing to the significant increase in differential abundance (*p* < 0.05) of the Proteobacteria. Within the Deltaproteobacteria, there were 22 families with a significant log_2_fold change (*p* < 0.05; Supplementary Table 2). Sixteen of these families increased, including six from the order Desulfuromonadales, and six families had a log_2_fold decrease (Supplementary Table 2). The order Desulfuromonadales includes members that are capable of anaerobic respiration, including iron-, sulfate-, and nitrate-reducing bacteria (Greene, 2014). The phylum Ignavibacteriae differentially increased in abundance (Figure 4D) with seven families significantly increasing (*p* < 0.05) all from the order Ignavibacteriales. Actinobacteria were significantly more abundant in the impacted amended microcosms with eight families significantly increasing (*p* < 0.05) and one family significantly decreasing in abundance (Figure 4D and Supplementary Table 2).

As was observed in the background microcosms, Verrucomicrobia in the impacted samples also decreased in relative abundance from day 0 sediment and unamended microcosms in comparison with the three amended microcosms. Within the Verrucomicrobia, five families had a significant log_2_fold decrease (*p* < 0.05, Supplementary Table 2). The majority of taxa that decreased in differential abundance (Figure 4D) in the amended microcosms were members of candidate phyla (Elusimicrobia, Parcubacteria, Omnitrophica, Deinococcus-Thermus, Peregrinibacteria, and Absconditabacteria). As little is known about these candidate taxa, their decrease in abundance suggests a sensitivity to some component of OG wastewaters. However, the candidate phyla Lokiarchaeota, Modulibacteria, and Cloacimonetes increased in differential abundance, suggesting that these organisms are not sensitive to OG constituents.

## Conclusion

Oil and gas wastewaters are complex mixtures that reflect the high salinity of the formation and the chemicals used for HF, and releases of these fluids have the potential for adverse effects on the environment. In previous work, we documented the environmental effects of OG wastewater disposal activities at a UIC facility *via* changes in stream geochemistry and microbial community composition immediately downstream from the injection operation (Akob et al., 2016; Fahrenfeld et al., 2017; Orem et al., 2017). Downstream surface water was elevated in Cl, Na, Sr, and organic constituents that were consistent with signatures of OG wastewaters. With the use of 16S rRNA gene amplicon sequencing (Akob et al., 2016), metagenomics (Fahrenfeld et al., 2017), and anaerobic cultivation studies (Mumford et al., 2018), the effects of the OG wastewater disposal operations on stream ecology were observed *via* shifts in microbial community composition and activity. These shifts were associated with an increase in the potential for anaerobic metabolism and taxa well adapted to the altered geochemistry observed immediately downstream of the UIC disposal facility, highlighting changes in the ecology at the site.

In this study, we expand our understanding of impacts to streambed microbial ecology by studying microbial responses to synthetic OG wastewater brine and HF fluid additives (DBNPA and ethylene glycol) on aerobic sediment microbial activity and community dynamics. We found that addition of brine (elevated TDS) had the largest impact on aerobic respiration in sediment microcosms and was most strongly linked to changes seen in microbial community structure. These changes in activity and community structure were similar even in the presence of DBNPA, a biocide, and ethylene glycol, a scale inhibitor, suggesting that elevated TDS is a major driver of effects. This finding is consistent with previous studies that saw inhibition of aerobic biodegradation due to the high salinity of shale gas wastewaters (Kekacs et al., 2015; McLaughlin et al., 2016; Hanson et al., 2019). The effects of elevated TDS were seen for both upstream (background) and downstream (impacted) stream sediments despite the samples having differing *in situ* microbial communities.

We saw an increase in anaerobic activity (e.g., iron reduction and methanogenesis) and taxa in downstream sediment microcosms despite incubation under an oxic headspace. Interestingly, addition of DBNPA with brine was observed to inhibit iron reduction, similar to that seen in anoxic incubations performed by Mumford et al. (2018) with stream bed sediments from the same OG wastewater disposal facility. In that study, the biocides DBNPA and bronopol both inhibited iron reduction. The shift toward anaerobic metabolism was not unexpected, as metagenomic sequencing (Fahrenfeld et al., 2017) and amplicon-based community characterization (Akob et al., 2016) saw higher abundance of methanogenic functional genes and taxa, respectively, in downstream impacted sediments. This result suggests that the microbial community in the downstream impacted sediments had adapted to the geochemical conditions (e.g., elevated TDS and NVDOC) that resulted from exposure to OG wastewater releases and other activities at the UIC disposal site. Our study indicates that releases from an OG wastewater disposal facility have the potential to alter streambed microbial communities and biogeochemical processes. Our findings are consistent with those reported by Campa et al. (2019), who saw that stream water microbial communities in areas with active HF operations responded differently to inputs of DBNPA compared with samples from areas without HF. DBNPA and/or its degradation byproducts could have inhibitory effects on portions of the microbial community as reported for stream water microcosm experiments by Campa et al. (2019). Streambed microbial communities form a crucial link in aquatic food webs and play a vital role in biogeochemical cycling with alterations to these communities potentially disrupting overall ecosystem function. Further, microbial communities have the potential to serve as a “canary in the coal mine” when assessing if OG operations have influenced nearby waters. Together, these findings indicate that OG operations are affecting stream microbial communities and that inputs of OG constituents such as brine and/or HF additives can have implications for ecosystem functions. To expand on this work, future studies are needed to identify rates and pathways of HFF additive degradation. Our team and others are providing multiple consistent lines of evidence to indicate that microbial communities in streams can adapt to OG-related inputs over time. The varied approaches yielding similar results from multiple groups across different OG-affected systems and regions highlights the importance of continued field and laboratory research on the potential environmental effects of OG development.

## Data Availability Statement

Microcosm data for this article are available from Akob et al. (2021). Microbial sequence data are deposited in the NCBI Sequence Read Archive under BioProject no. PRJNA554671 and accession numbers SRR9691065 to SRR9691094.

## Author Contributions

DA, IC, AM, and WO: conceptualization. DA, AM, CH, AF, and MV: data curation. DA, AM, and CH: formal analysis. DA, IC, AM, AF, MV, and WO: methodology and resources. DA, AM, AF, and MV: investigation. DA, AM, CH, and AF: visualization. DA, IC, AM, CH, and MV: writing—original draft and writing—review and editing. All authors contributed to the article and approved the submitted version.

## Conflict of Interest

The authors declare that the research was conducted in the absence of any commercial or financial relationships that could be construed as a potential conflict of interest. Any use of trade, product, or firm names is for descriptive purposes only and does not imply endorsement by the U.S. Government. The authors declare no competing financial interest.

## Publisher’s Note

All claims expressed in this article are solely those of the authors and do not necessarily represent those of their affiliated organizations, or those of the publisher, the editors and the reviewers. Any product that may be evaluated in this article, or claim that may be made by its manufacturer, is not guaranteed or endorsed by the publisher.

## Footnotes

1
www.fracfocus.org
