# Supplementary material for: Oil and Gas Wastewater Components Alter Streambed Microbial Community Structure and Function
Source: Front Microbiol. 2021 Nov 29;12:752947. doi: 10.3389/fmicb.2021.752947 (PMC8686200; doi:10.3389/fmicb.2021.752947)
Supplement: Supplementary file 1 [file Data_Sheet_1.pdf]

Supplementary Material

Oil and Gas Wastewater Components Alter Streambed Microbial Community Structure and Function

Denise M. Akob[1,*], Adam C. Mumford[2], Andrea Fraser[2,#], Cassandra R. Harris[1], William Orem[1,] Matthew Varonka[1], and Isabelle M. Cozzarelli[1]

[1] U.S. Geological Survey, Geology, Energy & Minerals Science Center, Reston, VA 20192 USA

[2] U.S. Geological Survey, Water Mission Area, Reston, VA 20192 USA

*** Correspondence:** Dr. Denise M. Akob, dakob@usgs.gov

[#]Present address: Hawn Environmental Lab, University of Maryland Baltimore County, Baltimore, MD

Keywords: unconventional oil and gas production, wastewaters, class II injection, microbial activity, microbial communities

Running Title: OG Wastewater Affects Microbial Communities

Table of Contents

SUPPLEMENTARY METHODS ... 2

1.1 MOTHUR CODE ... 2
1.2 R ANALYSIS CODE ... 3

SUPPLEMENTARY FIGURES AND TABLES ... 9

1.3 SUPPLEMENTARY FIGURES ... 9
1.4 SUPPLEMENTARY TABLES ... 12

SUPPLEMENTAL REFERENCES ... 14

Supplementary Methods

1.1 mothur code

```
#assemble contigs from raw reads, set processors based on machine
being used
make.contigs(file=WVHF_HFD.files, processors=48)
summary.seqs()
#screen sequences, parameters here are for v4 region
screen.seqs(fasta=current, group=current, maxambig=1, optimize=start-
end-minlength-maxlength, criteria=90, maxhomop=8)
summary.seqs()
unique.seqs(fasta=current)
count.seqs(name=current, group=current)
summary.seqs(count=current)
#align sequences. set path to reference sequences as needed
align.seqs(fasta=current,
reference=~/Working_Silva_Files/silva.nr_v128_v4.align, flip=T)
summary.seqs(count=current)
#screen sequences after alignment, parameters here are for v4
screen.seqs(fasta=current, count=current, optimize=start-end-
minlength-maxlength, criteria=95)
summary.seqs(count=current)
filter.seqs(fasta=current, vertical=T, trump=.)
summary.seqs(count=current)
unique.seqs(fasta=current, count=current)
#pre cluster sequences, parameter for v4
pre.cluster(fasta=current, count=current, diffs=3)
summary.seqs(count=current)
#KILL THE CHIMERAS!!!!!!!!!
chimera.vsearch(fasta=current, count=current, dereplicate=t)
#remove chimeric sequences from fasta file
remove.seqs(fasta=current, accnos=current)
summary.seqs(count=current)
#classify sequences
classify.seqs(fasta=current, count=current,
reference=~/Working_Silva_Files/silva.nr_v128_v4.align,
taxonomy=~/Working_Silva_Files/silva.nr_v128.tax, cutoff=80, probs=F)
remove.lineage(fasta=current, count=current, taxonomy=current,
taxon=Chloroplast-Mitochondria-unknown-Eukaryota)
summary.seqs(count=current)
#Call OTUs and classify them
cluster.split(fasta=current, count=current, taxonomy=current,
splitmethod=classify, taxlevel=5, cutoff=0.03, method=opti,
processors=48)
make.shared(list=current, count=current, label=0.03)
classify.otu(list=current, count=current, taxonomy=current,
label=0.03)
#basic pre-analysis of diversity
count.groups(shared=current)
collect.single(shared=current, calc=chao-invsimpson, freq=100)
rarefaction.single(shared=current, calc=sobs, freq=100)
```

```
summary.single(shared=current, calc=nseqs-coverage-sobs-invsimpson, subsample=T)
```

1.2 R analysis code

```
WVHF_HFD_seqs <- import_mothur(mothur_shared_file = "F:/WVHF_HFD_10-2017/original_files/WVHF_HFD_R_02_2018/WVHF_HFD_noSingle_NoUnclass.shared", mothur_constaxonomy_file = "F:/WVHF_HFD_10-2017/original_files/WVHF_HFD_R_02_2018/WVHF_HFD.nr_v128.wang.pick.pick.taxonomy")
WVHF_HFD_input <- import_mothur(mothur_shared_file = "F:/WVHF_HFD_4-2017/WVHF_HFD.shared", mothur_constaxonomy_file = "F:/WVHF_HFD_4-2017/WVHF_HFD.0.03.cons.taxonomy")
WVHF_HFD_tree <- read.tree("F:/WVHF_HFD_4-2017/WVHF_HFD_4-2017_otus/ExaML_result.WVHF_HFD_OTUs_ExaMLTree")
WVHF_HFD_treed <- merge_phyloseq(WVHF_HFD_input, WVHF_HFD_tree)
WVHF_HFD_metadata <- data.frame(read.csv("F:/WVHF_HFD_4-2017/WVHF_HFD_treatment_data.csv", header = TRUE, row.names = 1))
WVHF_HFD_Sample_Data <- sample_data(data.frame(Site = WVHF_HFD_metadata["Site"], Treatment = WVHF_HFD_metadata["Treatment"], Day = WVHF_HFD_metadata["Day"]))
WVHF_HFD_All <- merge_phyloseq(WVHF_HFD_treed, WVHF_HFD_Sample_Data)
WVHF_HFD_ALL_noSingletons_NoUnClassified <- subset_taxa(WVHF_HFD_All_noSingletons, Rank2!="Bacteria_unclassified")
WVHF_HFD_CLEAN <- WVHF_HFD_ALL_noSingletons_NoUnClassified
WVHF_HFD_CLEAN_D14 <- subset_samples(WVHF_HFD_ALL_noSingletons_NoUnClassified, Day == 14)
rarefied_WVHF_HFD_CLEAN_D14_otu_table <- as.data.frame(t(otu_table(rarefied_WVHF_HFD_CLEAN_D14)))
wvhf_hfd_chao1 <- estimateR(rarefied_WVHF_HFD_CLEAN_D14_otu_table, permutations=1000)
WVHF_HFD_CLEAN_D14_IMPACTED_Salt_Data <- read.csv("F:/WVHF_HFD_10-2017/original_files/WVHF_HFD_R_02_2018/WVHF_HFD_CLEAN_D14_Impacted_Salt_Data.csv", header = TRUE, row.names = 1)
wunifrac_WVHF_HFD_CLEAN_D14_IMPACTED <- phyloseq::distance(WVHF_HFD_CLEAN_D14_IMPACTED, "wunifrac")
unifrac_wvhf_hfd_clean_d14 <- UniFrac(WVHF_HFD_CLEAN_D14, weighted = TRUE)
wunifrac_WVHF_HFD_CLEAN_D14_BACKGROUND <- phyloseq::distance(WVHF_HFD_CLEAN_D14_BACKGROUND, "wunifrac")
WVHF_HFD_CLEAN_D14_BACKGROUND_Data <- data.frame(sample_data(WVHF_HFD_CLEAN_D14_BACKGROUND))
WVHF_HFD_CLEAN_D14_IMPACTED <- subset_samples(WVHF_HFD_CLEAN_D14, Site=="Impacted")
WVHF_HFD_CLEAN_D14_IMPACTED_Salt_Data <- read.csv("F:/WVHF_HFD_10-2017/original_files/WVHF_HFD_R_02_2018/WVHF_HFD_CLEAN_D14_Impacted_Salt_Data.csv", header = TRUE, row.names = 1)
wunifrac_WVHF_HFD_CLEAN_D14_IMPACTED <- phyloseq::distance(WVHF_HFD_CLEAN_D14_IMPACTED, "wunifrac")
#Rarefied, NMDS followed by Adonis(2)
```

```
#Background, comparing treatment with Brine to Unamended
HFD_Background_Alldata <- read.csv("F:/WVHF_HFD_10-
2017/original_files/WVHF_HFD_R_02_2018/WVHF_HFD_CLEAN_D14_Background_S
alt_Data.csv", header = TRUE, row.names = 1)
HFD_Background_Treatment_Data <- HFD_Background_Alldata[-c(10:12), ]
WVHF_HFD_CLEAN_D14_BACKGROUND_rarefied <-
rarefy_even_depth(WVHF_HFD_CLEAN_D14_BACKGROUND_NoZero, sample.size =
min(sample_sums(WVHF_HFD_CLEAN_D14_BACKGROUND_NoZero)), rngseed =
1981, trimOTUs = TRUE, replace = TRUE )
wunifrac_HFD_Background_rarefied <-
phyloseq::distance(WVHF_HFD_CLEAN_D14_BACKGROUND_rarefied, "wunifrac")
adonis_wunifrac_HFD_Background_rarefied <-
adonis2(wunifrac_HFD_Background_rarefied ~ Salt, )
nmds_wunifrac_HFD_Background_rarefied <-
metaMDS(wunifrac_HFD_Background_rarefied, k = 2, try = 1000, trymax =
1000)
ordiplot(nmds_wunifrac_HFD_Background_rarefied)
text(nmds_wunifrac_HFD_Background_rarefied, display = "sites")
with(WVHF_HFD_Background_AllData,
ordiellipse(nmds_wunifrac_HFD_Background_rarefied, Salt, scaling =
"symmetric", label = TRUE, kind = "ehull"))
HFD_Background_rarefied_Treatments <-
subset_samples(WVHF_HFD_CLEAN_D14_BACKGROUND_rarefied, Treatment !=
"Unamended")
sample_data(HFD_Background_rarefied_Treatments)
HFD_Background_rarefied_treatments_noZero <-
prune_taxa(taxa_sums(HFD_Background_rarefied_Treatments)>0,
HFD_Background_rarefied_Treatments)
wunifrac_HFD_Background_rarefied_treatments_noZero <-
phyloseq::distance(HFD_Background_rarefied_treatments_noZero,
"wunifrac")
nmds_wunifrac_HFD_Background_rarefied_treatments_noZero <-
metaMDS(wunifrac_HFD_Background_rarefied_treatments_noZero, k = 2, try
= 1000, trymax = 10000)
ordiplot(nmds_wunifrac_HFD_Background_rarefied_treatments_noZero)
text(nmds_wunifrac_HFD_Background_rarefied_treatments_noZero, display
= "sites")
with(HFD_Background_Treatment_Data,
ordiellipse(nmds_wunifrac_HFD_Background_rarefied_treatments_noZero,
Biocide, scaling = "symmetric", label = TRUE, kind = "ehull" ))
with(HFD_Background_Treatment_Data,
ordiellipse(nmds_wunifrac_HFD_Background_rarefied_treatments_noZero,
Biocide, scaling = "symmetric", label = TRUE, kind = "ehull" ))
with(HFD_Background_Treatment_Data,
ordiellipse(nmds_wunifrac_HFD_Background_rarefied_treatments_noZero,
Treatment, scaling = "symmetric", label = TRUE, kind = "ehull" ))
adonis_wunifrac_HFD_Background_Treatment_rarefied_biocide <-
adonis2(wunifrac_HFD_Background_rarefied_treatments_noZero~ Biocide,
HFD_Background_Treatment_Data)
adonis_wunifrac_HFD_Background_Treatment_rarefied_glycol <-
adonis2(wunifrac_HFD_Background_rarefied_treatments_noZero~ Glycol,
HFD_Background_Treatment_Data
```

```
#Impacted Site
HFD_Impacted_rarefied_Brine_Unam <-
subset_samples(HFD_Impacted_rarefied, Treatment == "Unamended" |
Treatment=="Brine")
wunifrac_HFD_Impacted_rarefied_Brine_Unam <-
phyloseq::distance(HFD_Impacted_rarefied_Brine_Unam, "wunifrac")
adonis_wunifrac_HFD_Impacted_rarefied_Brine_Unam <-
#Attempt with Family
HFD_Impacted_rarefied <-
rarefy_even_depth(WVHF_HFD_CLEAN_D14_IMPACTED_noZero, sample.size =
min(sample_sums(WVHF_HFD_CLEAN_D14_IMPACTED_noZero)), rngseed = 1981,
trimOTUs = TRUE, replace = TRUE))
wunifrac_HFD_Impacted_rarefied <-
phyloseq::distance(HFD_Impacted_rarefied, "wunifrac")
adonis_wunifrac_HFD_Impacted_rarefied <-
adonis2(wunifrac_HFD_Impacted_rarefied~Salt, HFD_Impacted_Alldata)
nmds_wunifrac_HFD_Impacted_rarefied <-
metaMDS(wunifrac_HFD_Impacted_rarefied, k = 2, try = 1000, trymax =
10000)
ordiplot(nmds_wunifrac_HFD_Impacted_rarefied)
text(nmds_wunifrac_HFD_Impacted_rarefied)
with(HFD_Impacted_Alldata,
ordiellipse(nmds_wunifrac_HFD_Impacted_rarefied, Salt, scaling =
"symmetric", label = TRUE, kind = "ehull"))
with(HFD_Impacted_Alldata,
ordiellipse(nmds_wunifrac_HFD_Impacted_rarefied, Treatment, scaling =
"symmetric", label = TRUE, kind = "ehull"))
HFD_Impacted_salt_DBNPA <- subset_samples(HFD_Impacted_rarefied,
Treatment= ("Brine") | ("DBNPA"))
HFD_Impacted_treatment <- subset_samples(HFD_Impacted_rarefied,
Treatment != "Unamended")
HFD_Impacted_treatment_rarefied_noZero <-
prune_taxa(taxa_sums(HFD_Impacted_treatment)>0,
HFD_Impacted_treatment)
wunifrac_HFD_Impacted_rarefied_treatments_noZero <-
phyloseq::distance(HFD_Impacted_treatment_rarefied_noZero, "wunifrac")
adonis_HFD_Impacted_rarefied_treatment_biocide <-
adonis2(wunifrac_HFD_Impacted_rarefied_treatments_noZero ~ Biocide,
HFD_Impacted_Treatment_Data)
adonis_HFD_Impacted_rarefied_treatment_glycol <-
adonis2(wunifrac_HFD_Impacted_rarefied_treatments_noZero ~ Glycol,
HFD_Impacted_Treatment_Data)
nmds_HFD_Impacted_rarefied_treatments <-
metaMDS(wunifrac_HFD_Impacted_rarefied_treatments_noZero, k = 2, try =
1000, trymax = 10000)
ordiplot(nmds_HFD_Impacted_rarefied_treatments)
text(nmds_HFD_Impacted_rarefied_treatments)
with(HFD_Impacted_Treatment_Data,
ordiellipse(nmds_HFD_Impacted_rarefied_treatments, Treatment, scaling
= "symmetric", label = TRUE, kind = "ehull"))
```

```
#DESeq2 Analysis
#Rank2
#Metadata table for DESeq2 code
```

Sample	Site	Treatment	Day	Labels	All_Params
BCK_Day_0_A	Background	Start	0	Background_Start	Background_Start
BCK_Day_0_B	Background	Start	0	Background_Start	Background_Start
BCK_Day_0_C	Background	Start	0	Background_Start	Background_Start
BCK_DBNPA_Day_14_A	Background	DBNPA	14	Background_DBNPA	Background_Treatment
BCK_DBNPA_Day_14_B	Background	DBNPA	14	Background_DBNPA	Background_Treatment
BCK_DBNPA_Day_14_C	Background	DBNPA	14	Background_DBNPA	Background_Treatment
BCK_Eth_Gly_Day_14_A	Background	Ethylene_Glycol	14	Background_Ethylene_Glycol	Background_Treatment
BCK_Eth_Gly_Day_14_B	Background	Ethylene_Glycol	14	Background_Ethylene_Glycol	Background_Treatment
BCK_Eth_Gly_Day_14_C	Background	Ethylene_Glycol	14	Background_Ethylene_Glycol	Background_Treatment
BCK_SALT_Day_14_A	Background	Brine	14	Background_Brine	Background_Treatment
BCK_SALT_Day_14_B	Background	Brine	14	Background_Brine	Background_Treatment
BCK_SALT_Day_14_C	Background	Brine	14	Background_Brine	Background_Treatment
BCK_UNAM_Day_14_A	Background	Unamended	14	Background_Unamended	Background_Unamended
BCK_UNAM_Day_14_B	Background	Unamended	14	Background_Unamended	Background_Unamended
BCK_UNAM_Day_14_C	Background	Unamended	14	Background_Unamended	Background_Unamended
IMP_Day_0_A	Impacted	Start	0	Impacted_Start	Impacted_Start
IMP_Day_0_B	Impacted	Start	0	Impacted_Start	Impacted_Start
IMP_Day_0_C	Impacted	Start	0	Impacted_Start	Impacted_Start
IMP_DBNPA_Day_14_A	Impacted	DBNPA	14	Impacted_DBNPA	Impacted_Treatment
IMP_DBNPA_Day_14_B	Impacted	DBNPA	14	Impacted_DBNPA	Impacted_Treatment
IMP_DBNPA_Day_14_C	Impacted	DBNPA	14	Impacted_DBNPA	Impacted_Treatment
IMP_Eth_Gly_Day_14_A	Impacted	Ethylene_Glycol	14	Impacted_Ethylene_Glycol	Impacted_Treatment
IMP_Eth_Gly_Day_14_B	Impacted	Ethylene_Glycol	14	Impacted_Ethylene_Glycol	Impacted_Treatment
IMP_Eth_Gly_Day_14_C	Impacted	Ethylene_Glycol	14	Impacted_Ethylene_Glycol	Impacted_Treatment
IMP_SALT_Day_14_A	Impacted	Brine	14	Impacted_Brine	Impacted_Treatment
IMP_SALT_Day_14_B	Impacted	Brine	14	Impacted_Brine	Impacted_Treatment
IMP_SALT_Day_14_C	Impacted	Brine	14	Impacted_Brine	Impacted_Treatment
IMP_UNAM_Day_14_A	Impacted	Unamended	14	Impacted_Unamended	Impacted_Unamended
IMP_UNAM_Day_14_B	Impacted	Unamended	14	Impacted_Unamended	Impacted_Unamended
IMP_UNAM_Day_14_C	Impacted	Unamended	14	Impacted_Unamended	Impacted_Unamended
MC_Blank_A	Blank	Blank	0	Blank	Blank
MC_Blank_B	Blank	Blank	0	Blank	Blank

```
wvhfd_phyloseq <- import_mothur(mothur_shared_file =
"WVHF_HFD.shared", mothur_constaxonomy_file =
"WVHF_HFD.0.03.cons.taxonomy")
wvhfd_metadata2 <- data.frame(read.csv("WV_Metadata2.csv", header =
TRUE, row.names = 1))
sample_data(wvhfd_metadata2)
wvhfd_metadata2_Sample_Data <- sample_data(data.frame(Site =
wvhfd_metadata2["Site"], Treatment = wvhfd_metadata2["Treatment"], Day
= wvhfd_metadata2["Day"], Labels = wvhfd_metadata2["Labels"],
All_Params = wvhfd_metadata2["All_Params"] ))
wvhfd_full_phyloseq <- merge_phyloseq(wvhfd_phyloseq,
wvhfd_metadata2_Sample_Data)
wvhfd_full_phyloseq_taxa <- tax_glom(wvhfd_full_phyloseq, taxrank =
"Rank2")
wvhfd_deseq2 <- phyloseq_to_deseq2(wvhfd_full_phyloseq_taxa,
~All_Params)
```

```
gm_mean = function(x, na.rm=TRUE) {
  exp(sum(log(x[x > 0]), na.rm = na.rm) / length(x))}
geoMeans <- apply(counts(wvhfd_deseq2), 1,gm_mean)
wvhfd_deseq2 = estimateSizeFactors(wvhfd_deseq2, geoMeans = geoMeans)
run_dds_wvhfd_deseq2 <- DESeq(wvhfd_deseq2, fitType = "local")
#Impacted treatments vs unamended
contrast_data <- results(run_dds_wvhfd_deseq2, contrast =
c("All_Params", "Impacted_Treatment", "Impacted_Unamended"))
contrast_data <- contrast_data[order(contrast_data$pvalue,na.last =
NA), ]
alpha <- 0.5
sigtab <- contrast_data[(contrast_data$pvalue < alpha), ]
sigtab <- cbind(as(sigtab, "data.frame"),
as(tax_table(wvhfd_full_phyloseq_taxa)[rownames(sigtab), ], "matrix"))
write.csv(sigtab, "IMP_Treatment-vs-Unamended_Phylum.csv")
#Background treatments vs unamended
contrast_data <- results(run_dds_wvhfd_deseq2, contrast =
c("All_Params", "Background_Treatment", "Background_Unamended"))
contrast_data <- contrast_data[order(contrast_data$pvalue,na.last =
NA), ]
alpha <- 0.5
sigtab <- contrast_data[(contrast_data$pvalue < alpha), ]
sigtab <- cbind(as(sigtab, "data.frame"),
as(tax_table(wvhfd_full_phyloseq_taxa)[rownames(sigtab), ], "matrix"))
write.csv(sigtab, "BCK_Treatment-vs-Unamended_Phylum.csv")
#Rank5
wvhfd_phyloseq <- import_mothur(mothur_shared_file =
"WVHF_HFD.shared", mothur_constaxonomy_file =
"WVHF_HFD.0.03.cons.taxonomy")
wvhfd_metadata2 <- data.frame(read.csv("WV_Metadata2.csv", header =
TRUE, row.names = 1))
sample_data(wvhfd_metadata2)
wvhfd_metadata2_Sample_Data <- sample_data(data.frame(Site =
wvhfd_metadata2["Site"], Treatment = wvhfd_metadata2["Treatment"], Day
= wvhfd_metadata2["Day"], Labels = wvhfd_metadata2["Labels"],
All_Params = wvhfd_metadata2["All_Params"] ))
wvhfd_full_phyloseq <- merge_phyloseq(wvhfd_phyloseq,
wvhfd_metadata2_Sample_Data)
wvhfd_full_phyloseq_taxa <- tax_glom(wvhfd_full_phyloseq, taxrank =
"Rank5")
wvhfd_deseq2 <- phyloseq_to_deseq2(wvhfd_full_phyloseq_taxa,
~All_Params)
gm_mean = function(x, na.rm=TRUE) {
  exp(sum(log(x[x > 0]), na.rm = na.rm) / length(x))}
geoMeans <- apply(counts(wvhfd_deseq2), 1,gm_mean)
wvhfd_deseq2 = estimateSizeFactors(wvhfd_deseq2, geoMeans = geoMeans)
run_dds_wvhfd_deseq2 <- DESeq(wvhfd_deseq2, fitType = "local")
#Impacted treatments vs unamended
contrast_data <- results(run_dds_wvhfd_deseq2, contrast =
c("All_Params", "Impacted_Treatment", "Impacted_Unamended"))
contrast_data <- contrast_data[order(contrast_data$pvalue,na.last =
NA), ]
```

```
alpha <- 0.5
sigtab <- contrast_data[(contrast_data$pvalue < alpha), ]
sigtab <- cbind(as(sigtab, "data.frame"),
as(tax_table(wvhfd_full_phyloseq_taxa)[rownames(sigtab), ], "matrix"))
write.csv(sigtab, "IMP_Treatment-vs-Unamended_Family.csv")
#Background treatments vs unamended
contrast_data <- results(run_dds_wvhfd_deseq2, contrast =
c("All_Params", "Background_Treatment", "Background_Unamended"))
contrast_data <- contrast_data[order(contrast_data$pvalue,na.last =
NA), ]
alpha <- 0.5
sigtab <- contrast_data[(contrast_data$pvalue < alpha), ]
sigtab <- cbind(as(sigtab, "data.frame"),
as(tax_table(wvhfd_full_phyloseq_taxa)[rownames(sigtab), ], "matrix"))
write.csv(sigtab, "BCK_Treatment-vs-Unamended_Family.csv")
```

Supplementary Figures and Tables

1.3 Supplementary Figures

Figure S1. Map of sampling sites in a stream running adjacent to a class II disposal facility in West Virginia. Site 4 is the upstream background site while site 7 is downstream of the injection well and adjacent to the former impoundment ponds and is affected by activities at the site. Modified from (Akob et al., 2016). The blue line highlights the stream, and the yellow outline is the location of the former impoundment ponds.

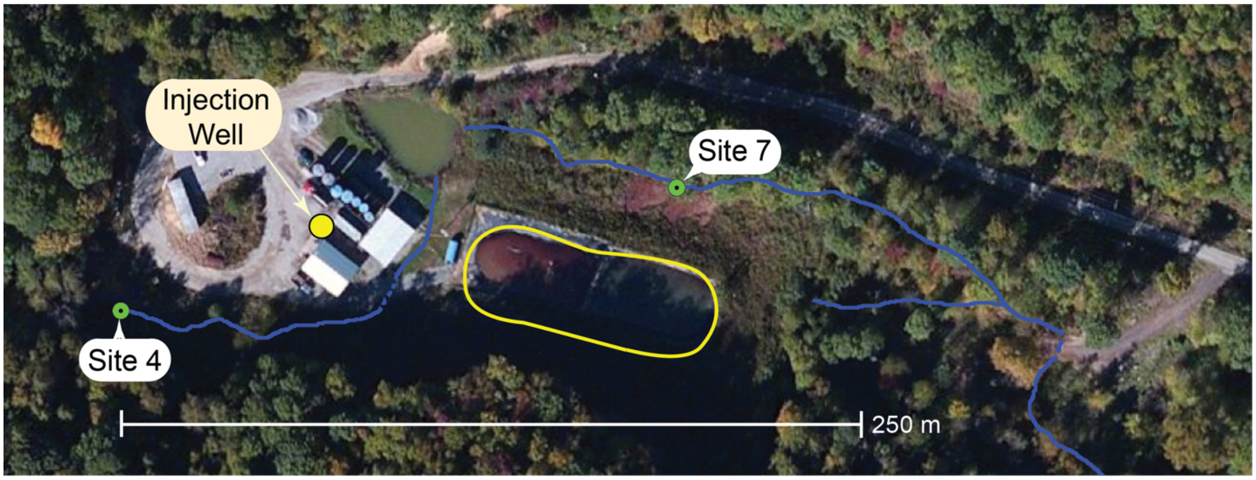

Figure S2: Concentrations of nonvolatile dissolved organic carbon (NVDOC) in (A) background and (B) impacted microcosms. Microcosms were amended with brine or shale gas production additives DBNPA (biocide) or ethylene glycol in brine, brine alone, or left unamended as controls. Synthetic brine mimicking the conditions of Marcellus shale produced water was added. Results are averages ± standard deviations for triplicate microcosms.

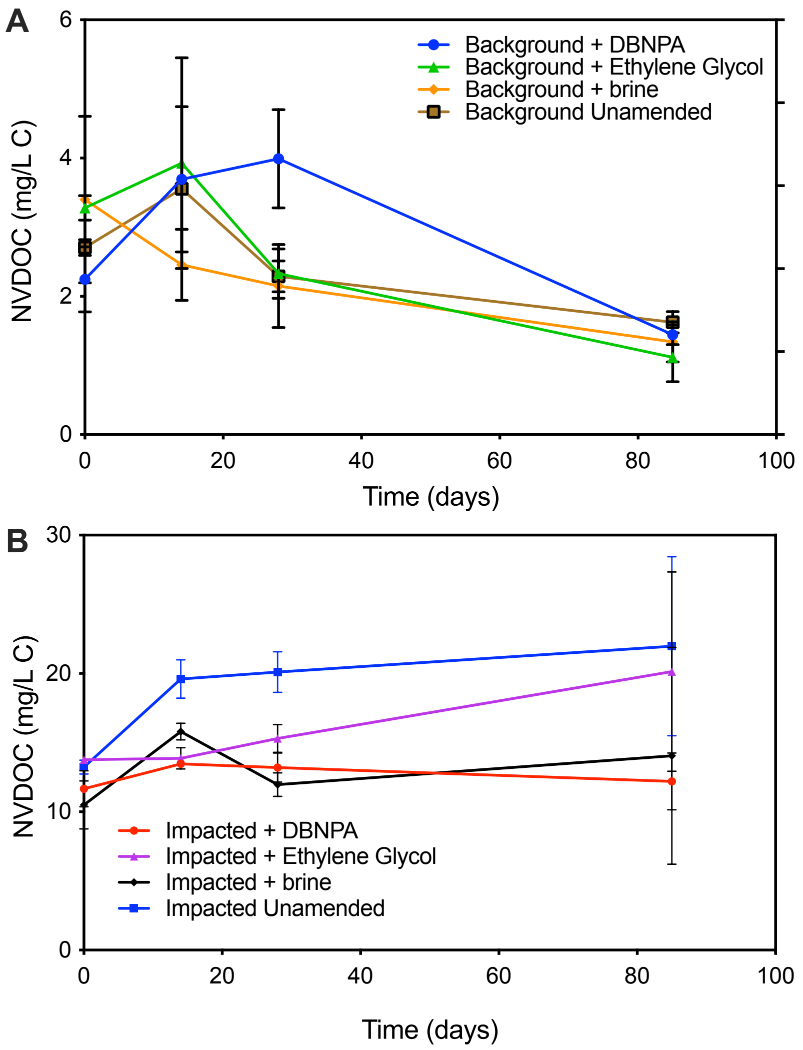

Figure S3: Oxygen concentrations in killed (A) background and (B) impacted microcosms. Microcosm treatments were amended with brine or shale gas production additives DBNPA (biocide) or ethylene glycol in brine, brine alone, or left unamended as controls. Synthetic brine mimicking the conditions of Marcellus shale produced water was added. Results are averages ± standard deviations for triplicate microcosms. Additional geochemical data from the killed control microcosms are presented in Akob et al. (2021).

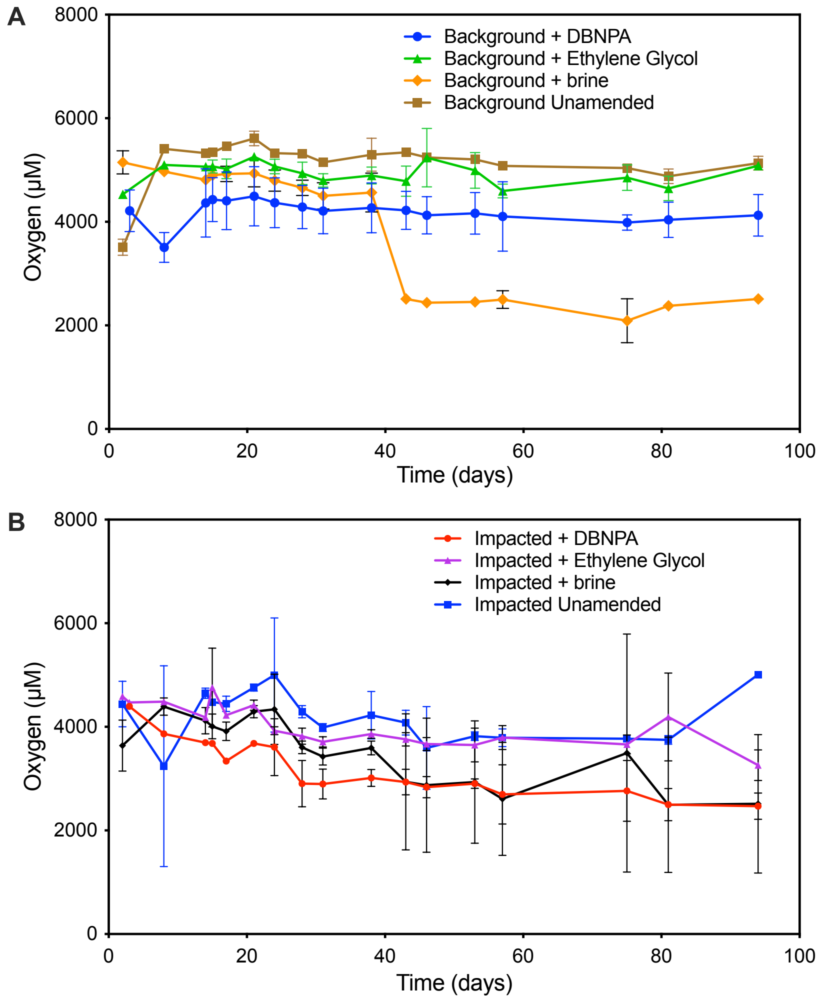

1.4 Supplementary Tables

Table S1. Concentrations of ethylene glycol in microcosms amended with brine + ethylene glycol.

Sample ID	Sediment Source	Sampling Day			
		Day 0	Day 14	Day 28	Day 57
HFD-11	Background	2.5	<d.l.[2]	<d.l.	<d.l.
HFD-12	Background	2.3	<d.l.	0	0
HFD-14	Impacted	2.9	0[3]	<d.l.	0
HFD-15	Impacted	3.1	<d.l.	0	<d.l.
HFD-17	Background (killed)[1]	4.3	3.9	n.a.[4]	0
HFD-19	Impacted (killed)	4.9	1.5	n.a.	1.5

[1] Killed control microcosms

[2] d.l. indicates the concentration was below the level of reliable quantification (1 mg/L)

[3] Zero indicates no ethylene glycol detected

[4] n.a. indicates no sample measured

Supplemental References

Akob, D.M., Mumford, A.C., Fraser, A., Harris, C.R., Baesman, S.M., Orem, W., et al. (2021). Data on the Effects of Oil and Gas Wastewater Components on Microbial Community Structure and Function. *U.S. Geological Survey data release*. doi: https://doi.org/10.5066/P9EJGL5R.

Akob, D.M., Mumford, A.C., Orem, W.H., Engle, M.A., Klinges, J.G., Kent, D.B., et al. (2016). Wastewater disposal from unconventional oil and gas development degrades stream quality at a West Virginia injection facility. *Environmental Science & Technology* 50(11), 5517-5525. doi: http://dx.doi.org/10.1021/acs.est.6b00428.
